# Immune-cell-guided nanozymes: redefining targeted therapy for neuroinflammation in multiple sclerosis

**DOI:** 10.1097/MS9.0000000000004858

**Published:** 2026-03-31

**Authors:** Minahil Tariq, Subahat Huma, Muhammad Talha, Mahnoor Fatima

**Affiliations:** a Department of Medicine, Nawaz Sharif Medical College, Gujrat, Pakistan; b Department of Medicine, King Edward Medical University, Lahore, Pakistan; c Department of Medicine, Shaheed Ziaur Rahman Medical College, Rajshahi Medical University, Bogura, Bangladesh

*To the Editor,*

Multiple sclerosis (MS) is primarily an autoimmune demyelinating disorder, exhibiting neurodegenerative features. It is characterized by oligodendrocyte loss, inflammation, and axonal injury^[^1^]^. In 2020, an estimated 2.8 million people experienced clinical manifestations of this disease, which rose to 131.12 Australians per 100 000 people in 2021. Women are affected 2–5 years earlier than men, and the mean diagnostic age is 32 years^[^2^]^. Affected individuals experience cognitive impairment, sensory impairment, and vision loss, depending on the area of white matter affected. Neuro-inflammation in the MS process produces reactive oxygen species (ROS), which then mediates myelin phagocytosis by macrophages. The therapeutic effect of nanozymes involves sequestration of macrophages, thereby downregulating the severity of the disease. Current treatment strategies effectively palliate the symptoms and suffering of the patient. Sphingosine 1-phosphate receptor modulators, anti-CD20 monoclonal antibodies, and anti-CD52 monoclonal antibodies face resistance in crossing the blood–brain barrier (BBB) due to their complex structure and low permeability^[^3^]^.

Nanozymes are nanomaterials having enzyme-like catalytic properties. Their redox activity sequesters oxygen and nitrogen species, targeting oxidative stress and attenuating mitochondrial decay^[^1^]^. Physiochemical properties of nanomaterials, such as size, shape, morphology, and composition, provide a compelling alternative in improving symptoms of the patients^[^3^]^. Nanoparticles enable drug delivery with precision, strong physical and chemical stability, high drug-loading capacity, and low systemic side effects^[^4^]^. This protective therapy scavenges ROS by its enzyme-mimetic activity, thereby preserving oligodendrocytes and inhibiting cellular attrition. Cerium-oxide-based nanoparticles contribute to neuroprotection and antioxidant activity, a hallmark pathology in neurodegenerative diseases like MS, Alzheimer’s disease, Huntington’s disease, Parkinson’s disease^[^3^]^, and ischemic stroke^[^1^]^.

This method consists of *ex vivo* harvesting of the patient’s monocytes, loading them with antioxidant nanozymes, which are engineered catalytic nanoparticles that mimic the body’s antioxidant enzymes, and then returning them to the body. Nanozymes have the capability to neutralize the free radicals and thus lower the oxidative stress, which is one of the main factors causing neuroinflammation to develop. For example, related systems of single-atom nanozymes (which are delivered by microneedles) have shown around 70% of continuous release of the nanozyme amount over 48 h in the conditions that are similar to those of human body, thus making the ROS elimination efficient and the neuroinflammation at the lesion sites remitting. This has resulted in a considerable reduction of behavioral disorders and visible symptoms (including those related to movement) in preclinical models of Parkinson’s disease plagued with motor-sided problems. Nanozymes have already been explored for use in other neuroinflammatory situations, but their association with monocyte delivery systems can be considered a large conceptual step forward in the case of MS. The monocytes that have been reintroduced would, by the action of chemokine gradients and inflammatory adhesion signals, naturally move to the active MS plaques, delivering the nanozyme payload across the BBB and directly to the places where demyelination and inflammation occur^[^5^]^.

The use of the “Trojan horse” method in the treatment of MS through a 2021 research study is one of the important applications mentioned in the past medical literature. This method employed the LIFNano-CD4 nanoparticles that were aimed at CD4+ T cells for brain delivery and loaded with the leukemia inhibitory factor (LIF). Testing was done in the Biozzi ABH relapsing-remitting MS mouse model, where the LIFNano-CD4 (1.0 mg/mouse) was injected intracranially once, and the clinical scores were found to be significantly lower in comparison to the controls. Besides, the reduction in clinical scores was shown in all four groups of 12 mice each, thus indicating dose-dependent neuroprotection, myelin preservation, and a shift toward the regulatory T cells, which resulted in 1000-fold potency gains without toxic effects. This monocyte-like strategy is an indication of the targeted antioxidant delivery to inflamed plaques and also facilitates the *ex vivo* nanozyme-loading proposals^[^6^]^.

To illustrate, preclinical studies have looked into targeting monocyte-directed nanotherapy in MS models. In a mouse experiment with autoimmune encephalomyelitis, curcumin-loaded high-density lipoprotein-mimicking peptide–phospholipid scaffolds were selectively absorbed by the inflammatory monocytes and their crossing over the BBB was considerably slowed down. This treatment brought down clinical morbidity in mice from 100% to only 30%, thus demonstrating the power of monocyte-based “Trojan horse” nanodelivery in inflammation control of the central nervous system (CNS) and bettering disease outcomes^[^7^]^.

The mentioned targeting strategy gives an active approach, and thus it solves passive nanocarrier delivery’s disadvantages: (1) larger specificity – monocytes are naturally predisposed to penetrate the CNS lesions; (2) efficient crossing of the BBB, thus avoiding a significant obstacle to direct drug delivery into the inflamed tissue, and (3) lower systemic exposure and decreased side effects, as the payload is kept in the autologous cells until it is released at the specific site. Besides, nanozymes can be designed to combat oxidative stress, which is gaining more and more recognition as a factor contributing to MS lesion development and neurodegeneration; thus, immune-guided targeting is combined with catalytic therapeutic action efficiently^[^8^]^.

Monocyte-mediated nanozyme delivery is an attractive concept, but it still has to bridge a number of translational difficulties. There is no consensus on the protocols to isolate and load monocytes *in vitro*, so questions over the scalability, cost, and reproducibility of the process are raised. It is uncertain how the altered monocyte phenotype or viability after manipulation would affect CNS trafficking or inflammatory behavior; in any case, such an influence might be unpredictable. In addition, long-term biodistribution, off-target accumulation, and immunogenicity of nanozymes, as well as regulatory issues for cell-based nanotherapies, have been characterized only to a limited extent, thus posing a barrier to clinical application^[^9^]^.

In short, the combination of new nanocatalytic therapeutics with immune-cell-guided delivery, in this case, indirectly points to the extension of the existing paradigms of MS therapy, which previously focused merely on symptom alleviation, to the intervention of lesions specifically. The accumulation of evidence shown invites, in a way, the improvement of the already existing strategies for the crossing of the active BBB, the designation of oxidative stress as a modifiable target, the refining of *ex vivo* monocyte engineering, proof by means of meticulously considered translational models, and the synchronization of nanotherapy development with precision immunology. These viewpoints, in unison, indicate the necessity to change the prevailing ideas about passive drug delivery, to encourage interdisciplinary collaboration, and to hasten, under strict monitoring, the clinical trials of biologically intelligent nanotherapeutic platforms for demyelinating diseases.

This letter is in line with the TITAN Guidelines on the need for transparency in AI use in healthcare^[^10^]^.

## Data Availability

Not applicable to this study.
